# Accuracy of HIV Risk Perception in East Zimbabwe 2003–2013

**DOI:** 10.1007/s10461-018-2374-0

**Published:** 2018-12-19

**Authors:** Robin Schaefer, Ranjeeta Thomas, Constance Nyamukapa, Rufurwokuda Maswera, Noah Kadzura, Simon Gregson

**Affiliations:** 1 Department of Infectious Disease Epidemiology, Imperial College London, Norfolk Place, London, W2 1PG UK; 2 Department of Health Policy, London School of Economics and Political Science, Houghton Street, London, WC2A 2AE UK; 3 Biomedical Research and Training Institute, Harare, Zimbabwe

**Keywords:** HIV prevention, Risk perception, HIV incidence, Accuracy of perceptions, Sexual risk

## Abstract

Risk perception for HIV infection is an important determinant for engaging in HIV prevention behaviour. We investigate the degree to which HIV risk perception is accurate, i.e. corresponds to actual HIV infection risks, in a general-population open-cohort study in Zimbabwe (2003–2013) including 7201 individuals over 31,326 person-years. Risk perception for future infection (no/yes) at the beginning of periods between two surveys was associated with increased risk of HIV infection (Cox regression hazard ratio = 1.38 [1.07–1.79], adjusting for socio-demographic characteristics, sexual behaviour, and partner behaviour). The association was stronger among older people (25+ years). This suggests that HIV risk perception can be accurate but the higher HIV incidence (1.27 per 100 person-years) illustrates that individuals may face barriers to HIV prevention behaviour even when they perceive their risks. Gaps in risk perception are underlined by the high incidence among those not perceiving a risk (0.96%), low risk perception even among those reporting potentially risky sexual behaviour, and, particularly, lack of accuracy of risk perception among young people. Innovative interventions are needed to improve accuracy of risk perception but barriers to HIV prevention behaviours need to be addressed too, which may relate to the partner, community, or structural factors.

**Electronic supplementary material:**

The online version of this article (10.1007/s10461-018-2374-0) contains supplementary material, which is available to authorized users.

## Introduction

HIV incidence remains high in many countries, particularly in sub-Saharan Africa, with reductions failing to meet international targets [1]. In part, this reflects continued low use of primary HIV prevention methods, including condoms, voluntary medical male circumcision (VMMC), and pre-exposure prophylaxis (PrEP) [2]. One factor that is considered important—often necessary—for motivation to engage in HIV prevention behaviour is perceiving a personal risk for HIV acquisition [3]. Associations have been found between HIV risk perception and delayed sexual debut [4], condom use [5, 6], and adherence to daily PrEP [7–9]. Given these links between risk perception and preventative behaviour, HIV prevention programmes frequently aim to raise awareness of risks and increase risk perception [2, 10]. Risk perception has also been proposed as the first step in early formulations of HIV prevention cascades [11], a framework to improve the planning, implementation, and evaluation of HIV prevention programmes and interventions. However, one common concern is that the lack of use of prevention methods, and thus continuing high HIV incidence, does not only reflect a widespread lack of risk perception but also a mismatch between actual and perceived risk of HIV infection—i.e. lack of accuracy of risk perception.

Even within generalised epidemics, HIV infection risk varies considerably across areas [12, 13] and within populations, with some groups, for example adolescent girls and young women [14], exhibiting disproportionally high HIV incidence. It is therefore vital that those with increased HIV infection risk perceive their risk and engage in protective behaviour, particularly because targeted HIV prevention activities may be more effective in reducing HIV incidence [15]. Nevertheless, while “unrealistic optimism”—underestimating one’s risk—has been demonstrated for HIV infection risk [16–18], evidence for a match between self-perceived and actual HIV infection risk is limited—despite the importance widely attached to HIV risk perception.

Current evidence comes largely from cross-sectional studies that are difficult to interpret [19–21]. Measuring accuracy of risk perception in terms of its association with actual HIV infection risk requires longitudinal data with objective measurement of HIV incidence. In a longitudinal study among injecting drug users in Canada, risk perception predicted HIV acquisition [22]. However, results from this high-risk population that is characterised by very high HIV incidence are not generalisable to settings with generalised epidemics. The only other previously published longitudinal study that analysed this association found that perceived risk in young South African women did not correspond to actual risk of acquiring HIV [23]. However, the study used self-reported HIV status to determine eligibility at baseline, so results may not be reliable. In this article, longitudinal data from a large, prospective HIV sero-survey, collected over a ten-year period of high HIV incidence, are used to measure accuracy of perceived risk of HIV infection in a representative sample of the population in Manicaland, east Zimbabwe.

## Methods

### Setting and Data

Data for this study were taken from the Manicaland General-Population Cohort Study (Manicaland Study) that was implemented in Manicaland, east Zimbabwe. In Manicaland, HIV prevalence declined from over 25% at the end of the 1990s to levels of about 11% in 2015–2016 [24], partially due to behaviour change [25, 26]. However, despite decreases from peaks of 1.8% in the mid-2000s, HIV incidence in the general population remains high at just under 1% for females and 0.5% for males [27]. Uptake of VMMC is low [11], and among young women, a target for PrEP in sub-Saharan Africa, sexual relationships with older men are common while condom use is limited [28]. Oral PrEP has recently become available in Zimbabwe through small-scale research and pilot projects, focusing largely on young female sex workers, leading to just over 3000 people being initiated on PrEP at the end of 2017 [29].

The Manicaland Study is a long-term general-population open-cohort study, with six surveys conducted in three districts since 1998. Each survey included a household census in 12 sites (eight in the most recent survey in 2012–2013) to identify participants. These were representative of the population in Manicaland that is characterised by different socio-economic strata, including small towns, subsistence farming areas, agricultural estates, and roadside business centres. Participants were prospectively followed in each survey but newly identified eligible individuals were included in surveys. Surveys included between 8000 and 15,000 adults aged 15–54 years. Participation rates varied between 73.0 and 79.5%. Periods between surveys were about 3 years and three attempts were made to reach participants for follow-up. Loss-to-follow-up resulted largely due to participants becoming ineligible through migrating out of the study area or death. Among those who remained eligible, follow-up ranged between 77.0 and 96.4%.

The Manicaland Study was originally set up to evaluate a cluster-randomised HIV prevention trial in the first two surveys but the research aims were expanded from survey round three to investigate the dynamics and determinants of the HIV epidemic in the area (we included only data from survey three for main analyses, see below). After survey two, data from the Manicaland Study was used to evaluate national HIV control programmes but the study itself did not implement interventions. Data collected in the Manicaland Study include HIV sero-testing, so HIV infection was objectively determined in this study, and information on demographic and socio-economic characteristics, sexual behaviour, and perceptions about HIV/AIDS. To reduce social desirability bias, informal confidential voting interview methods were used [30], in which participants answered more sensitive sexual behaviour questions on pieces of paper and inserted these into a box instead of responding directly to the interviewer. Ethical approval for the Manicaland Study was obtained from the Imperial College London Research Ethics Committee and the Medical Research Council of Zimbabwe. More details on the Manicaland Study are available elsewhere [27] and online (http://www.manicalandhivproject.org/).

### Data Analysis

The main analysis was restricted to survey rounds three (2003–2005) to six (2012–2013) because the survey question measuring risk perception was different in the first two survey rounds (“Do you think you could become infected with HIV yourself in the future?” in surveys one and two as opposed to “If you are not infected, do you think you are in danger of getting infected now or in the future?” from survey three). While the change in measurement may be small, the effects of this are unclear, so excluding survey rounds one and two was considered more conservative. Another reason for restricting the main analysis to data from survey three was that measurements of some other key variables were different or data were not collected in earlier surveys (including on condom use and sexual risk factors; see below). Nevertheless, in a secondary analysis, data from the first two surveys were included (see Supplementary Material, p. 5).

The risk perception measure allowed ‘yes’, ‘no’, and ‘don’t know’ responses. ‘Don’t know’ answers (9.6% over surveys three to six) were excluded from all analyses since these could not be categorised as either ‘yes’ or ‘no’, as described in the Supplementary Material (p. 3). To implement longitudinal analyses for capturing incident HIV infections and estimate HIV incidence, data were restricted to those who (1) participated in at least two surveys; (2) were HIV-negative at the beginning of the period between two surveys; and (3) those who reported having had sex at the beginning of inter-survey period since HIV is nearly exclusively sexually transmitted in the study population [31]. The beginning of the period between surveys refers to the interview date of the first of the two interviews of the survey pair. Individuals could contribute more than one survey pair by participating in more than two surveys but had to be HIV-negative at the beginning of each survey pair.

Those who started sexual activity during survey rounds were excluded because sexual debut is likely to have a strong influence on risk perception and other key variables were unavailable for those not sexually active. Trends in risk perception, potentially risky sexual behaviour, perceived risky behaviour of the partner, and condom use at the beginning of each period between surveys were described. This included data for survey six (the end of the final inter-survey period), as well as one (1998–2000) and two (2001–2003) to describe trends comprehensively, although these data were not included in the main regression analyses. A sexual risk variable was created based on the number of sexual risk factors (none, one, more than one), including multiple partners in the past 12 months, casual partners in the past 3 years, and concurrent sexual relationships at the moment. Perceived partner risky behaviour was based on reporting that the partner has other partners (partner concurrency). Condom use was based on reported use during last sexual intercourse.

Risk perception was tested for its longitudinal association with HIV acquisition as a measure of accuracy. Methods for estimating HIV incidence in the study data are described elsewhere [28]. In short, variables at the beginning of the period between surveys were tested for association with HIV infection in Cox regression. For those who turned HIV-positive between two surveys, the date of HIV infection was unknown, so 30 random infection dates between surveys were imputed and results for imputed data sets were pooled. This approach was chosen as using the mid-point date between surveys may introduce bias [32, 33]. Individuals were censored at their date of HIV infection or 55th birthday. Regression models controlled for age and sex (model 1); marital status, educational attainment, and household wealth index (model 2) (identified as important socio-demographic characteristics in preliminary analyses; see Supplementary Material, p. 4); and own sexual risk, partner risky behaviour, and condom use (model 3). Models were estimated separately including: (1) risk perception (no/yes); and (2) risk perception with reported reasons for perceiving an infection risk (multiple partners, partner has other partners, marrying someone who is HIV-positive, and ‘other’). Risk perception itself does not cause HIV infection; rather, any association between risk perception and HIV incidence reflects accurate recognition of other risk factors. Changes in the association between risk perception and HIV incidence in models 2 and 3 could provide insights into how risk perception was linked with HIV infection risk.

Sub-analyses tested for associations between risk perception and HIV acquisition risk (controlling for age and sex) in different time periods relating to the introduction of antiretroviral treatment (ART) (ART roll-out phase [2003–2008] and post-ART period [2009–2013]) and by sex, age group (15–24; 25–54 years), marital status, sexual risk (none; at least one risk factor), condom use, and perceived partner risk (partner had no other partners; had other partners). Interactions were also tested for in separate regression models including the socio-demographic or behavioural variable and an interaction term with risk perception.

All regressions included survey round and study site as covariates. The inclusion of these variables was important to account for any broader environmental, potentially time-varying factors that may confound the relationship between risk perception and HIV incidence. Study location-level (which meant village-level in most cases) cluster-robust standard error estimation was used. Proportional hazards assumptions were met (Supplementary Material, p. 6). All variables and their measurements are further described in the Supplementary Material (p. 2).

## Results

### Trends in HIV Risk Perception and Sexual Risk

**Fig. 1:**
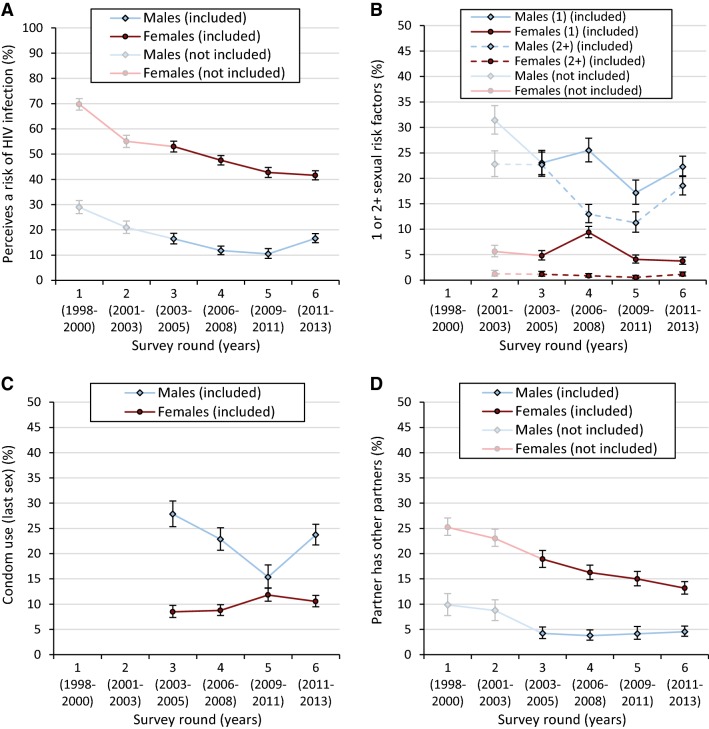
Trends in proportions and 95% confidence intervals of HIV risk perception and sexual behaviour by sex, Manicaland, Zimbabwe. **a** HIV risk perception (survey rounds 1–6); **b** number of sexual risk factors (available from survey round 2); **c** condom use during last sexual intercourse (available from survey round 3); and **d** reported partner concurrency (survey rounds 1–6). HIV risk perception was measured using a different question in survey rounds 1–2 and data from these rounds were not used in the main analysis for this study, so these data are indicated by the shaded points and lines. Data from survey 6 were included in the study but values of variables were not tested for association with HIV infection risk given that survey 6 was the end of the last inter-survey period

Over survey rounds three to six, 10,774 observations met the inclusion criteria for this study (67.0% female), based on 7201 individuals. 2830 individuals (39.3%) participated in more than two surveys and 743 (10.3%) participated in all four included surveys. Patterns of HIV risk perception by socio-demographic and behavioural characteristics are shown in Table 1. Among males (N = 3553), 13.0% (95% confidence interval [CI] = 11.9–14.1%) perceived a risk of HIV infection, and 47.5% (46.4–48.7%) among females (N = 7221), with declines over time observed for both sexes (Fig. 1a). For both sexes, risk perception was higher in those with sexual risk factors and in those reporting that their partners had other partners. However, even among those with two or more sexual risk factors, 44.8% (32.3–58.1%) of females (N = 60) and 77.1% (73.4–80.4%) of males (N = 556) reported that they do not perceive a risk of HIV infection. Similarly, 35.0% (32.3–37.8%) of females (N = 1172) and 78.0% (70.3–84.2%) of males (N = 141) who reported that their partners had other partners did not perceive a risk of HIV infection.

**Table 1:** HIV risk perception by socio-demographic and behavioural characteristics, Manicaland, Zimbabwe, 2003–2011

	Males (N = 3553)		Females (N = 7221)	
	N (%)	% perceives risk (95% CI)	N (%)	% perceives risk (95% CI)
Age				
15–24 years	790 (22.2)	17.8 (15.3–20.7)	1344 (18.6)	40.5 (37.9–43.1)
25–54 years	2763 (77.8)	11.6 (10.4–12.8)	5877 (81.4)	49.2 (47.9–50.4)
Marital status				
Never married	763 (21.5)	21.5 (18.7–24.6)	202 (2.81)	45.3 (38.5–52.3)
Married	2635 (74.4)	10.1 (9.00–11.3)	5673 (78.9)	50.8 (49.5–52.1)
Separated/divorced	116 (3.27)	19.8 (13.5–28.2)	494 (6.87)	40.0 (35.7–44.4)
Widowed	29 (0.82)	20.7 (9.12–40.4)	812 (11.4)	29.8 (26.7–33.0)
Education				
None/primary	966 (27.3)	11.0 (9.17–13.1)	3324 (46.7)	46.5 (44.8–48.2)
Secondary/higher	2571 (72.7)	13.7 (12.5–15.1)	3794 (53.3)	48.6 (47.0–50.2)
Wealth index quintile				
Poorest	493 (14.0)	12.6 (9.92–15.8)	1103 (15.4)	46.4 (43.5–49.4)
2nd poorest	1623 (45.9)	12.2 (10.7–13.8)	3545 (49.5)	45.9 (44.3–47.5)
3rd poorest	1052 (29.8)	14.1 (12.2–16.4)	1936 (27.0)	51.2 (49.0–53.4)
4th poorest	340 (9.62)	14.8 (11.4–19.0)	530 (7.40)	49.1 (44.9–53.4)
Least poor	25 (0.71)	4.00 (0.48–26.3)	45 (0.63)	44.4 (30.3–59.6)
Sexual risk factors^a^				
None	2175 (61.8)	9.17 (8.03–10.5)	6650 (92.9)	47.2 (46.0–48.4)
1	786 (22.4)	16.8 (14.4–19.6)	449 (6.27)	51.1 (46.5–55.7)
2+	556 (15.8)	22.9 (19.6–26.6)	60 (0.84)	55.2 (41.9–67.7)
Partner has other partners				
No	3381 (96.0)	12.6 (11.5–13.8)	5888 (83.4)	44.6 (43.4–45.9)
Yes	141 (4.00)	22.0 (15.8–29.7)	1172 (16.6)	65.0 (62.2–67.7)
Condom use during last sex				
No	2738 (77.5)	11.0 (9.83–12.2)	6489 (90.3)	47.5 (46.3–48.7)
Yes	793 (22.5)	20.0 (17.3–22.9)	697 (9.70)	48.1 (44.4–51.9)

Values represent the sample sizes (N) and relative sizes in percent (%) of the different categories of variables as well as the percentage of those in these categories perceiving a risk for HIV infection with 95% confidence intervals (95% CI). Values may not add up to 100% due to rounding. All statistics are based on the sample as used in the main analyses (i.e. data from the beginning of the period between surveys from survey round 3 to 6)

^a^The sexual risk variable was based on three variables: reporting more than one sexual partner in the past 12 months; reporting at least one casual partner in the past 3 years; and reporting concurrent sexual partner at the time of the survey

38.2% (36.6–39.8%) of males and 7.1% (6.6–7.8%) of females reported at least one sexual risk factor. For males, proportions reporting of risk factors declined over time but increased in the most recent survey (Fig. 1b); for females, there was no clear trend. Condom use was low in the population, with 22.5% (21.1–23.9%) of males and 9.7% (9.0–10.4%) of females reporting condom use during last sexual intercourse. For males, there was a marked decrease in condom use followed by a sharp increase in the most recent survey (Fig. 1c); while, for females, there was a slight increase over time. Risk perception was higher among males reporting condom use while there was no difference among females (Table 1). 4.0% (3.4–4.7%) of males and 16.6% (15.8–17.5%) of females reported that their partners had other partners, with a long-term decreasing trend for females (Fig. 1d).

### Accuracy of Risk Perception

343 new HIV infections occurred over 31,326 person-years. HIV incidence was similar in males (1.19 per 100 person-years [95% CI 0.99–1.40%]) and females (1.04% [0.90–1.18%]). HIV incidence among those who perceived a risk for HIV infection was 1.27% (1.06–1.48%) compared to 0.96% (0.83–1.10%) among those who did not (adjusted hazard ratio [aHR] = 1.34 [1.05–1.72], adjusted for age, sex, survey round, and study site). This roughly one-third higher risk was not markedly affected when controlling for other socio-demographic characteristics, own and partner sexual risk factors, or condom use (Table 2). The association was stronger among females (aHR = 1.48 [1.09–1.99]) than males (aHR = 1.28 [0.81–2.00]) (Table 3) (although the estimates for males and females were not significantly different and there was no significant interaction by sex: Table 4). Results were similar when including data from earlier survey rounds (model 1, both sexes combined: aHR = 1.36 [1.13–1.65]; Supplementary Material, p. 5), despite the changing risk perception measure.

**Table 2:** Risk perception and HIV incidence (both sexes combined), Manicaland, Zimbabwe, 2003–2013

Variable	N (%)	Inf/pyrs (IR)	Model 1 (n = 10,732)		Model 2 (n = 10,494)		Model 3 (n = 10,214)	
			aHR (95% CI)	p-value	aHR (95% CI)	p-value	aHR (95% CI)	p-value
Risk perception								
No	6857 (63.9)	191/19,884 (0.96)	1 (Reference)		1 (Reference)		1 (Reference)	
Yes	3879 (36.1)	144/11,348 (1.27)	1.34 (1.05–1.72)	0.021	1.41 (1.11–1.80)	0.005	1.38 (1.07–1.79)	0.014
Risk perception: reason								
No		191/19,884 (0.96)	1 (Reference)		1 (Reference)		1 (Reference)	
Yes: has multiple partners	121 (3.19)	16/354 (2.52)	3.88 (2.38–6.33)	< 0.001	3.66 (2.26–5.91)	< 0.001	3.30 (1.89–5.77)	< 0.001
Yes: partner has other partners	1244 (32.8)	51/3709 (1.38)	1.28 (0.87–1.91)	0.213	1.35 (0.90–2.03)	0.145	1.35 (0.87–2.08)	0.178
Yes: marry HIV–positive partner	210 (5.50)	20/640 (3.07)	2.34 (1.50–3.66)	< 0.001	2.32 (1.43–3.74)	< 0.001	2.34 (1.43–3.83)	< 0.001
Yes: other	2222 (58.5)	55/6407 (0.87)	0.96 (0.69–1.33)	0.803	1.05 (0.75–1.47)	0.771	1.05 (0.76–1.48)	0.763

Values are sample sizes (N) and percentages (%) for variable categories, new HIV infections (inf) per person-years (pyrs), crude incidence rates per 100 person-years (IR), adjusted hazard ratios (aHR), 95% confidence intervals (CI), and p-values. Different models estimated associations for risk perception (no/yes) (top panel) and risk perception by reason (bottom panel). Sample sizes and percentages for reasons for risk perception refer to the sample of those who perceived a risk. The covariate results are not shown. Regression results are based on 30 imputed random dates of HIV infection between surveys. Participants were censored at their 55th birthday. Sample sizes differ between the models due to missing data on variables included in the models

Model 1: age, sex, survey round, study site

Model 2: age, sex, marital status, educational attainment, household wealth index, survey round, study site

Model 3: age, sex, marital status, educational attainment, household wealth index, sexual risk factors, condom use (last sex), partner has other partners, survey round, study site

**Table 3:** Risk perception and HIV incidence by sex, Manicaland, Zimbabwe, 2003–2013

Variable	Males				Females			
	N (%)	Inf/pyrs (IR)	Model 3 (n = 3433)		N (%)	Inf/pyrs (IR)	Model 3 (n = 6781)	
			aHR (95% CI)	p-value			aHR (95% CI)	p-value
Risk perception								
No	3083 (87.0)	102/9287 (1.10)	1 (Reference)		3774 (52.5)	89/10,597 (0.84)	1 (Reference)	
Yes	460 (13.0)	24/1458 (1.66)	1.28 (0.81–2.00)	0.289	3419 (47.5)	120/9890 (1.21)	1.48 (1.09–1.99)	0.011
Risk perception: reason								
No		102/9287 (1.10)	1 (Reference)			89/10,597 (0.84)	1 (Reference)	
Yes: has multiple partners	52 (11.6)	8/158 (5.06)	3.34 (1.51–7.37)	0.003	69 (2.06)	8/196 (4.09)	3.17 (1.23–8.15)	0.017
Yes: partner has other partners	97 (21.7)	3/314 (0.96)	0.66 (0.15–2.86)	0.589	1147 (34.2)	48/3396 (1.42)	1.51 (0.95–2.40)	0.078
Yes: marry HIV-positive partner	114 (25.5)	8/371 (2.06)	1.77 (0.79–3.94)	0.165	96 (2.87)	12/268 (4.47)	2.70 (1.37–5.32)	0.004
Yes: other	184 (41.2)	6/572 (0.98)	0.84 (0.34–2.05)	0.701	2038 (60.8)	50/5835 (0.85)	1.24 (0.86–1.78)	0.257

Values are sample sizes (N) and percentages (%) for variable categories, new HIV infections (inf) per person-years (pyrs), crude incidence rates per 100 person-years (IR), adjusted hazard ratios (aHR), 95% confidence intervals (CI), and p-values. Different models estimated associations for risk perception (no/yes) (top panel) and risk perception by reason (bottom panel), for males and females separately. Sample sizes and percentages for reasons for risk perception refer to the sample of those who perceived a risk. The covariate results are not shown. Regression results are based on 30 imputed random dates of HIV infection between surveys. Participants were censored at their 55th birthday. Only results for model 3 are shown

Model 3: age, marital status, educational attainment, household wealth index, sexual risk factors, condom use (last sex), partner has other partners, survey round, study site

**Table 4:** Risk perception and HIV incidence by socio-demographic characteristics and behaviour, Manicaland, Zimbabwe, 2003–2013

Variable	Inf/pyrs (IR)	Hazard ratio of HIV infection when perceiving a risk (vs no risk perception)			p-value of interaction
		N	aHR	95% CI	
Sex					
Males	128/10,774 (1.19)	3543	1.27	(0.82–1.99)	
Females	215/20,562 (1.05)	7193	1.41	(1.07–1.85)	0.723
Age group (years)^a^					
15–24	89/6585 (1.35)	2134	1.08	(0.69–1.70)	
25–54	255/24,751 (1.03)	8602	1.58	(1.19–2.10)	0.644
Marital status^b^					
Never married	39/3104 (1.26)	964	2.05	(1.04–4.05)	
Currently married	237/24,029 (0.99)	8282	1.29	(0.94–1.76)	
Formerly married	64/4079 (1.58)	1447	1.54	(0.92–2.57)	0.079
Time period^c^					
ART roll-out	276/23,062 (1.20)	7384	1.44	(1.10–1.89)	
Post-ART	68/8274 (0.83)	3352	1.25	(0.74–2.11)	0.722
Sexual risk					
No risk factor	239/25,377 (0.94)	8794	1.41	(1.07–1.87)	
At least one risk factor	99/5689 (1.74)	1849	1.18	(0.75–1.88)	0.694
Condom use (last sex)					
No use	276/26,672 (1.04)	9200	1.17	(0.88–1.56)	
Used condom	67/4493 (1.48)	1479	2.58	(1.61–4.13)	< 0.001
Partner has other partners					
No	282/26,939 (1.05)	9238	1.38	(1.06–1.80)	
Yes	56/3853 (1.45)	1307	1.00	(0.53–1.89)	0.950

The table shows for each sub-group for each variable the number of new HIV infections (inf) per person-years (pyrs) and crude incidence rates per 100 person-years (IR). For each of these sub-groups, Cox regression models were implemented to test for the association between HIV risk perception and HIV infection risk, with adjusted hazard ratios (aHR) and 95% confidence intervals (CI) referring to the ratio of perceiving a risk (vs not perceiving a risk). Sample sizes (N) refer to the samples for the regression for each sub-group. Each regression model included age and sex as additional variables. A higher aHR suggest that the association between risk perception and HIV infection was stronger in that sub-group, thus suggesting higher accuracy. This interaction was tested in separate models that included the socio-demographic or behavioural variable and an interaction term of this variable with risk perception; the p-values refer to this interaction

^a^Age (continuous) was not included as a covariate in analyses of age groups

^b^Those divorced/separated and those widowed were grouped together into the ‘formerly married’ category. The p-value of the interaction term is for the interaction as a whole, not between specific categories

^c^Survey round was not included as a covariate in the analyses by time period. The ART roll-out period refers to the inter-survey periods of survey 3 (2003–2005) to 4 (2006–2008) and 4 to 5 (2009–2011). The post-ART period refers to the inter-survey period of survey 5 to 6 (2012–2013)

Excluding ‘other’ reasons, suspecting that the partner had other partners was the most common reason for HIV risk perception among females; men were more likely to state having multiple partners as the reason for risk perception, although suspecting partner concurrency and marrying an HIV-infected person were more common reasons (Table 3). Risk perception was associated with increased HIV infection risk regardless of the reason (excluding ‘other’ reasons) (Table 2), although to varying degree. Controlling for socio-demographic characteristics and own and partner sexual behaviour, HIV infection risk was 230% higher among those who perceived a risk because they had multiple partners compared to those not perceiving a risk (aHR = 3.30 [1.89–5.77]) (similar for both sexes, Table 3), but only 35% higher in those perceiving a risk because they thought their partner had other partners (aHR = 1.35 [0.87–2.08]). Those perceiving a risk because they might marry a partner who is HIV-infected were also at greater risk of HIV infection (aHR = 2.34 [1.43–3.83]).

When stratifying by socio-demographic and behavioural characteristics, the general trend of higher HIV infection risk among those perceiving a risk was seen in most sub-groups, although with varying strength (Table 4). The strength of the association—so the accuracy of HIV risk perception—was higher among those who were older and those who had never been married, during the ART roll-out phase, in those without sexual risk factors, reporting that their partner had no other partners, and who used a condom during last sexual intercourse. However, sample sizes in some sub-groups were small and interaction terms in were not statistically significant, except for marital status and condom use (Table 4).

## Discussion

In this large general-population cohort in east Zimbabwe, sexually active individuals who perceived a risk of future HIV infection had a one-third greater risk of acquiring HIV infection than those who did not, accounting for a range of socio-demographic and behavioural characteristics as well as potential time-varying and broader environmental confounders. This represents the first scientifically robust evidence from a general-population sample in a generalised HIV epidemic that HIV risk perception can be accurate. Accurate risk perception is vital so that individuals who are actually at increased risk of HIV infection also perceive themselves to be at risk and thus are motivated to protect themselves against infection.

The relationship between behaviour, perceptions, and HIV infection risk is complex. Someone who engages in behaviours associated with increased risk of HIV infection (e.g. having multiple or non-regular partners [34–36]) but uses protective measures (e.g. condoms) may not perceive a risk for HIV infection. This may be accurate if condoms are used consistently, but individuals may actually still be at an increased risk if condoms are used only some of the time. An advantage of this study was that it used biomarkers for HIV infection to objectively determine HIV infection risks. We therefore considered the outcome of behaviours and it was not necessary to know each individual’s behaviour for making conclusions about the accuracy of perceptions. With this approach, we demonstrate significant gaps in risk perception. Many individuals did not perceive a risk despite engaging in potentially high-risk behaviour. 45% of females and 80% of males reporting two or more sexual risk factors did not report that they were at risk of HIV infection. While engaging in these behaviours is not inherently ‘risky’, we show that HIV incidence was high (1%) in individuals who did not perceive themselves to be at risk, thus these individuals did not accurately evaluate their HIV infection risks. Furthermore, while the higher HIV infections risk among those who perceive a risk demonstrates the accuracy of these perceptions, it also underlines that these individuals may face barriers preventing them from translating this perception into protective behaviour. In fact, if they engaged in protective behaviour, they may not have reported risk perception (although risk perception was higher among males who used condoms).

The observed relationship between risk perception and HIV incidence differed markedly across sub-groups, although risk perception tended to be associated with higher incidence in all groups. The relationship was stronger among those who were older and was weak among those aged under 25. Therefore, on average, young people who perceived and who did not perceive a risk were at the same risk of HIV infection, so risk perception did not correspond to increased risk of HIV infection. This does not mean that every young person was at the same risk of HIV infection; rather, many young people at increased infection risk did not perceive this increased risk and some young people not at increased risk perceived themselves to be at risk. This leads to inappropriate patterns of motivation to engage in HIV prevention, which is of concern since HIV incidence was generally higher in younger people, particularly young women [28].

The association between risk perception and HIV incidence was stronger in those who had not yet married than in currently married people. This may be because never married people had only short-term partners, so they only need to evaluate their own behaviour, not the risk resulting from their long-term partners, and those who engage in risky behaviours are aware of their risks. This is further supported by the strong association between risk perception and HIV incidence when one’s own risky behaviour is given as the reason. Individuals who reported that their partners had other partners were more likely to perceive a risk for HIV infection; however, the relationship between risk perception and infection risk was weak among those reporting risk perception because their partners had other partners. This may be because there are more possible sources of error when assessing infection risks from the partner as opposed to one’s own behaviour, as there may be errors in assessing whether or not the partner actually has other partners and in assessing the risk associated with these partners. HIV risk perception was more strongly associated with HIV incidence in people who used condoms than in those who did not. Our measure of condom use was based on use during last sexual intercourse and therefore, in most cases, probably represents condom use with regular partners. The relatively high accuracy of risk perception in this group may be because many of these individuals know or have good reason to suspect that their partners are HIV-positive, but, again, the high HIV incidence underscores that these individuals failed to adequately protect themselves against HIV infection.

This study analysed the association between risk perception and HIV infection risk completely relying on biomarkers for HIV infection, differing from a study in South Africa that excluded individuals at baseline (in 2005) based on self-reported HIV status and that did not find an association between HIV infection risk and HIV risk perception [23]. In 2005, HIV testing was likely to be uncommon (30% of South Africans were ever tested in 2005 [37]), so participants may already have been unknowingly infected with HIV at baseline, which could have introduced significant noise into the data. Despite this, the results of the two studies are not inconsistent as the South African study was limited to young women and we also found low accuracy of risk perception in this group in east Zimbabwe. The results of the current study may be more generalisable to other parts of sub-Saharan Africa, however, since patterns of marriage and sexual behaviour are probably more representative [38] than those from the metropolitan area of Cape Town, South Africa. The considerable decline in HIV incidence in Zimbabwe over time is unlikely to limit the generalisability of the findings to other settings with more moderate declines in incidence given that accuracy of risk perception does not necessarily depend on background levels of incidence and populations across sub-Saharan Africa have been extensively exposed to HIV prevention messages and programmes, although it is unclear whether these may have been more successful in improving accuracy of HIV risk perception in Zimbabwe.

Reported risk perception has been declining over time in the study population. To the degree that individuals accurately recognise their risks, declining risk perception may reflect declines in reported sexual risk factors (among males) and suspecting that the partner has other partners (among females), and indirectly the decline in HIV incidence. The increase in risk perception among males in the most recent survey round also corresponds to an increase in risk behaviour. The increasing availability of ART may have further contributed to reductions in perceived risk. The association between risk perception and HIV incidence was weaker in the post-ART period compared to the ART roll-out phase, possibly because ART attenuates risks of HIV infection, making risk perceptions less accurate—e.g. sexual intercourse with an HIV-positive partner may be perceived as risky but is actually not associated with an increased risk if the partner is on ART. In this context of declining risk perception, and possibly reduced accuracy of risk perception, it is worrying that men’s condom use declined until the most recent survey and that women’s condom use remained low. Even in the post-ART period, HIV incidence has been high (0.83%) (which, as an average, masks heterogeneity in incidence among different population groups), with ART coverage still below 40% in the 2012–2013 survey [27]. However, statistical power for these sub-analyses was limited and interactions were not statistically significant in most cases.

While HIV incidence was measured objectively, this study relied on self-reports for other variables. Due to social desirability bias, risk perception may be under-reported to avoid being associated with risky behaviour. This may partly explain the high HIV incidence among those not reporting risk perception, so the difference in incidence between those who did and did not perceive a risk may be underestimated, making our findings conservative. Similarly, sexual risk behaviour may be under-reported, despite the informal confidential voting interview methods to reduce social desirability bias [30]. Inaccurate measurement of sexual behaviour may also explain why the association between risk perception and HIV incidence did not markedly change when controlling for sexual risk factors. If these risk factors had been perfectly measured, the strength of the association between risk perception and incidence would likely have been affected as risk perception is associated with HIV infection risk through the recognition of these sexual risk factors. However, while reported levels of risk perception and risky sexual behaviour may be biased, observed trends are unlikely to be affected by this. Another limitation is the simple binary measure for risk perception. While this measure refers to future HIV infection—in contrast to other studies that only considered perceptions of current infection status [39]—it does not permit investigation of whether different levels of risk perception are associated with different levels of HIV incidence.

Despite limitations in the data, this study demonstrates that subjective perceptions of HIV infection risk can be accurate, and so supports HIV prevention programmes aiming at increasing risk perception. At the same time, the higher HIV incidence among those perceiving a risk underlines the considerable barriers to engaging in HIV prevention behaviour individuals may face even if they recognise their risks, which may be beyond the individual’s control [40]. This includes partner refusal—which is important for condom use as well as adherence to PrEP [41] and uptake of VMMC [42]—social norms [43], and structural barriers [44], including those relating to the legal system. This study supports calls to increase attention towards HIV prevention [45] given the continuing high HIV incidence in this population and declines and considerable gaps in risk perception—despite long-term exposure to HIV prevention programmes. The variation in accuracy of risk perception across sub-groups is also a cause of concern—particularly the low accuracy of risk perception among young people and the difficulties in determining exposure to risks from the partner compared to one’s own behaviour. This underscores the need for innovative approaches to improve risk perception such as the recent application of methods from behavioural economics to correct risk perception in South African teenagers [46]. However, given the broad range of factors influencing HIV prevention behaviour, as is increasingly recognised in approaches to HIV prevention [1, 43, 44, 47–49], interventions focusing on increasing risk perception must be accompanied by other interventions to strengthen motivation for using prevention methods, access to these methods—including removing structural barriers—and individual capacity for effective use of these, which may involve partner-based interventions [50].

## Electronic supplementary material

Below is the link to the electronic supplementary material.

Supplementary material 1 (PDF 320 kb)
