# Supplementary material for: Accuracy of HIV Risk Perception in East Zimbabwe 2003–2013
Source: AIDS Behav. 2018 Dec 19;23(8):2199–209. doi: 10.1007/s10461-018-2374-0 (PMC6647479; doi:10.1007/s10461-018-2374-0)
Supplement: Supplementary file 1 — Supplementary material 1 (PDF 320 kb) [file 10461_2018_2374_MOESM1_ESM.pdf]

Supplemental Material

Accuracy of HIV Risk Perception in East Zimbabwe 2003-13

Table of contents for the supplemental material

Additional information on data and variables........ 2
Analyses of excluded responses for risk perception........ 3
Preliminary analyses for background characteristics........ 4
Analyses for all survey rounds (1998-2013)........ 5
Proportional hazards assumption........ 6

Additional information on data and variables

In this section, data underlying analysed variables is discussed, unless variables are self-explanatory (e.g. age).

Risk perception

- *Risk perception:* In the main article that includes data from survey round 3 (2003-2005), risk perception was based on answers to the question "If you are not infected, do you think you are in danger of getting infected now or in the future?" with "yes", "no", and "don't know" response possibilities. In the two surveys before 2003 (considered in a sensitivity analysis, see below), risk perception was measured with "Do you think you could become infected with HIV yourself in the future?" with the same response possibilities. Participants responding with "don't know" where excluded, as discussed further below.
- *Risk perception reasons:* Reasons for perceiving a risk for HIV infection were determined with the question "Why do you think you might become infected". The response categories "regular partner had many partners" and "future partner may have other partners" were grouped together in the main analyses.

Background characteristics

- *Education:* As there very few individuals with no or with higher education, two categories of educational attainment were created: 'no or primary education' and 'secondary or higher education'.
- *Marital status:* Marriage was defined as a long-term relationships that lasted for at least 12 months.
- *Socio-economic status:* A wealth index variable was created to represent socio-economic status. This was based on characteristics of and items present in the household, so it does not directly measure individual wealth. The index was based on sellable assets like cars and non-sellable assets like the water source; this index ranged from zero to one and was divided into quintiles.

Sexual behaviour and risk factors

- *Sexual risk factors:* The sexual risk factor variable used in the study was an index based on the number of positive responses to the three separate variables of multiple sexual partners, casual sexual partners, and concurrent sexual partners. The variable on casual sexual partners was only asked from survey round 2 (2001-3), so the sexual risk index was not available for survey round 1 (as shown in Figure 1 in the main article).
    - *Multiple sexual partners:* Reporting more than one sexual partner for the question "How many different sexual partners have you had in the last 12 months?"
    - *Casual sexual partners:* Reporting at least one non-regular partner for the question "How many different non-regular sexual partners have you had in the last three years?"
    - *Concurrent sexual partnerships:* Reporting more than one sexual relationship for the question "How many sexual relationships do you consider yourself to be involved in at the moment?"
- *Partner concurrency:* Partner concurrency was based on whether the respondent reported at least one other partner for the question "If you took a guess, how many partners other than yourself (and any co-wives) do you think your current spouse/partner has had in the last 12 months?"
- *Condom use:* Condom use was based on the question "Did you use condoms throughout the last time you have sex?" that was asked from survey round 3 (2003-5). During earlier survey rounds condom use was measured only for the past two weeks, which is not directly comparable, so the variables were not combined in Figure 1 in the main article.

Analyses of excluded responses for risk perception

Participants responding “don’t know” to the survey question on risk perception were excluded from all analyses. This includes 132 males (3.59% of male sample) and 1013 females (12.3%). As outlined in Table S1, this group includes a diverse set of individuals that could not easily be grouped together with either those perceiving a risk or those not perceiving a risk. For males, those responding with “don’t know” were similar to those not perceiving a risk in terms of age and marriage patterns, but they were different to those who did and those who did not perceive a risk in terms of sexual behaviour. Females reporting “don’t know” were also dissimilar with regard to sexual risk factors to those perceiving and those not perceiving a risk. The diversity of this group is further underlined by the association with HIV incidence (model 1, aHR=1.26 [0.92-1.74]), which is between the no risk perception and risk perception estimate. The small sample size of the “don’t know” category and thus larger uncertainty as well as the fact that it is unclear whether this group should be classified more like the no risk perception or the risk perception group meant that it was excluded from the main analyses.

Table S1: Socio-demographic and behavioural characteristics in the study sample by risk perception response and sex, Manicaland, Zimbabwe, 2003-2013.

	Male			Females		
	Perceives risk (N=460)	Does not perceive risk (N=3083)	Don’t know (N=132)	Perceives risk (N=3419)	Does not perceive risk (N=3774)	Don’t know (N=1013)
Age						
15-24 years	30.7	21.1	13.6	15.9	21.2	19.6
25-54 years	69.4	79.0	86.4	84.1	78.8	80.4
Marital status						
Never married	35.8	19.5	19.7	2.67	2.93	2.57
Married	57.9	76.8	75.8	84.5	74.1	82.0
Separated/divorced	5.02	3.02	4.6	5.76	7.82	7.62
Widowed	1.31	0.75	0.00	7.11	15.2	7.81
Education						
None/primary	23.1	28.0	26.4	45.7	47.8	51.5
Secondary/higher	76.9	72.0	73.6	54.4	52.2	48.5
Wealth index quintile						
Poorest	13.5	14.1	15.4	15.1	15.8	14.3
2nd poorest	43.0	46.4	36.9	47.7	51.2	47.8
3rd poorest	32.3	29.3	34.6	29.0	25.2	30.9
4th poorest	10.9	9.40	10.8	7.62	7.18	6.34
Least poor	0.22	0.78	2.31	0.59	0.67	0.69
Sexual risk factors						
None	43.5	64.6	64.6	92.3	93.45	93.7
1	28.8	21.4	14.6	6.76	5.85	4.78
2+	27.7	14.0	20.8	0.94	0.70	1.49
Partner has other partners						
No	93.4	96.5	95.4	77.2	89.5	88.4
Yes	6.64	3.54	4.62	22.8	10.5	11.6
Condom use during last sex						
No	65.4	79.3	75.0	90.3	90.5	91.6
Yes	34.6	20.7	25.0	9.72	9.51	8.42

Values are percentages of the overall sample for males and females. Values may not add up to 100% due rounding.

Preliminary analyses for background characteristics

Socio-demographic and economic background characteristics that could confound the relationship between risk perception and HIV incidence need to be associated with both HIV incidence and risk perception. Each variable was tested separately for association with HIV incidence in Cox regression models and for association with risk perception in logistic regression models (Table S2), controlling for age, sex, survey round, and study site. For associations with risk perception, data were analysed as panel, accounting from correlation of several observations per participant.

Being currently enrolled in school was not further considered as the association with risk perception was confounded by marital status (not shown). Distance to town and religion do not show strong associations with either risk perception or HIV incidence, so were not further considered.

Table S2: Socio-demographic characteristics in association with HIV incidence and risk perception, Manicaland, Zimbabwe, 2003-2013.

	Association with HIV incidence (Cox regression)			Association with risk perception (logistic regression)		
	aHR	95% CI	p-value	aOR	95% CI	p-value
Marital status						
Never married	1 (Reference)			1 (Reference)		
Married	1.39	(0.88-2.19)	0.162	0.67	(0.55-0.82)	<0.001
Separated/divorced	2.99	(1.74-5.14)	<0.001	0.48	(0.37-0.63)	<0.001
Widowed	2.30	(1.17-4.53)	0.016	0.29	(0.21-0.38)	<0.001
Education						
None/primary	1 (Reference)			1 (Reference)		
Secondary/higher	1.60	(1.23-2.08)	<0.001	1.09	(0.99-1.22)	0.092
School enrolment						
No	1 (Reference)			1 (Reference)		
Yes	0.68	(0.33-1.41)	0.305	1.45	(0.99-2.13)	0.055
Wealth index quintile						
Poorest	1 (Reference)			1 (Reference)		
2nd poorest	1.62	(1.14-2.30)	0.007	0.97	(0.84-1.11)	0.639
3rd poorest	1.32	(0.92-1.93)	0.147	1.19	(1.01-1.39)	0.032
4th poorest	1.25	(0.78-2.00)	0.355	1.12	(0.92-1.37)	0.257
Least poor	1.38	(0.46-4.13)	0.563	0.79	(0.48-1.31)	0.359
Distance to town						
0-4km	1 (Reference)			1 (Reference)		
5-9km	0.74	(0.48-1.13)	0.158	0.86	(0.72-1.02)	0.083
10+ km	0.83	(0.59-1.16)	0.282	1.03	(0.88-1.19	0.728
Religion						
Christian mission churches	1 (Reference)			1 (Reference)		
Apostolic churches	0.87	(0.68-1.12)	0.289	0.95	(0.69-1.30)	0.736
Traditional	0.34	(0.08-1.41)	0.137	0.92	(0.82-1.05)	0.214
Other	0.68	(0.48-0.95)	0.022	0.92	(0.81-1.06)	0.271
No religion stated	0.95	(0.66-1.35)	0.768	1.17	(0.98-1.41)	0.086

For associations with HIV incidence, values are adjusted hazard ratios (aHR), 95% confidence intervals (CI), and p-values. For associations with risk perception, values are adjusted odds ratios (aOR), 95% confidence intervals (CI), and p-values. Each variable was tested in a separate model, controlling for age, sex, survey round, and study site (covariate results not shown). Results for Cox regressions are based on 30 imputed random dates of HIV infection between surveys and participants were censored at their 55th birthday.

Analyses for all survey rounds (1998-2013)

In the main paper, analyses on the relationship between risk perception and HIV infection risk were restricted to data from survey 3 (2003-5) due to the change in the wording of the risk perception question and data for some variables only being available from survey 3. In Table S3, the results for Cox regression models testing for an association between risk perception and HIV infection risk for both sexes combined and by sex are presented for all six survey rounds (1998-2013), controlling for age (and sex), survey round, and study site (referred to as model 1 in the main article). The results are very similar to those presented in the main article that used data from 2003-5.

Table S3: Risk perception and HIV incidence, Manicaland, Zimbabwe, 1998-2013.

	Both sexes combined Model 1 (n=16175)			Males Model 1 (n=5779)			Females Model 1 (n=10396)		
**Variable**	**Inf/pyrs (IR)**	**aHR (95% CI)**	**p**	**Inf/pyrs (IR)**	**aHR (95% CI)**	**p**	**Inf/pyrs (IR)**	**aHR (95% CI)**	**p**
Risk perception									
No	301/27362 (1.10)	1 (Reference)		171/13691 (1.25)	1 (Reference)		130/13671 (0.95)	1 (Reference)	
Yes	272/18166 (1.50)	1.36 (1.13-1.65)	0.001	63/2961 (2.14)	1.42 (1.03-1.96)	0.030	209/15205 (1.37)	1.37 (1.10-1.71)	0.005

Values are new HIV infections (inf) per person-years (pyrs), crude incidence rates per 100 person-years (IR), adjusted hazard ratios (aHR), 95% confidence intervals (CI), and p-values. The covariate results are not shown. Results are based on 30 imputed random dates of HIV infection between surveys. Participants were censored at their 55th birthday. Sample sizes for different models due to missing data on variables included in the models.
Model 1: Age, sex, survey round, study site

Proportional hazards assumption

Cox regression assumes proportional hazards. Each variable in this study was used in a univariate Cox regression (mid-points of dates between survey rounds were used as HIV infection dates), and plots of scaled Schoenfeld residuals against time were produced. In addition, generalised linear regression of scaled Schoenfeld residuals over time were tested for nonzero slopes. The null hypothesis of these tests was a zero slope and a significant result would indicate deviation from the proportional hazards assumption. Table S4 presents these tests by sex and Figure S1 presents the Schoenfeld plots by sex for the risk perception variables. For no variable a significant deviation from the proportional hazards assumption is indicated. The lowest p-values are for males for the risk perception variables, but the Schoenfeld plots do not suggest significant deviation from the proportional hazards assumption.

Table S4: Testing for nonzero slopes of Schoenfeld residuals over time by sex (results are for global tests for variables with more than two categories).

	Males	Females
	p-value	p-value
Risk perception	0.0732	0.5764
Risk perception by reason	0.0816	0.7830
Age group (5 years)	0.3727	0.8054
Marital status	0.9219	0.4034
Education	0.1630	0.2287
Wealth index quintile	0.3907	0.1042
Sexual risk factors	0.6306	0.5579
Partner concurrency	0.7468	0.9784
Condom use (last sex)	0.4390	0.7173

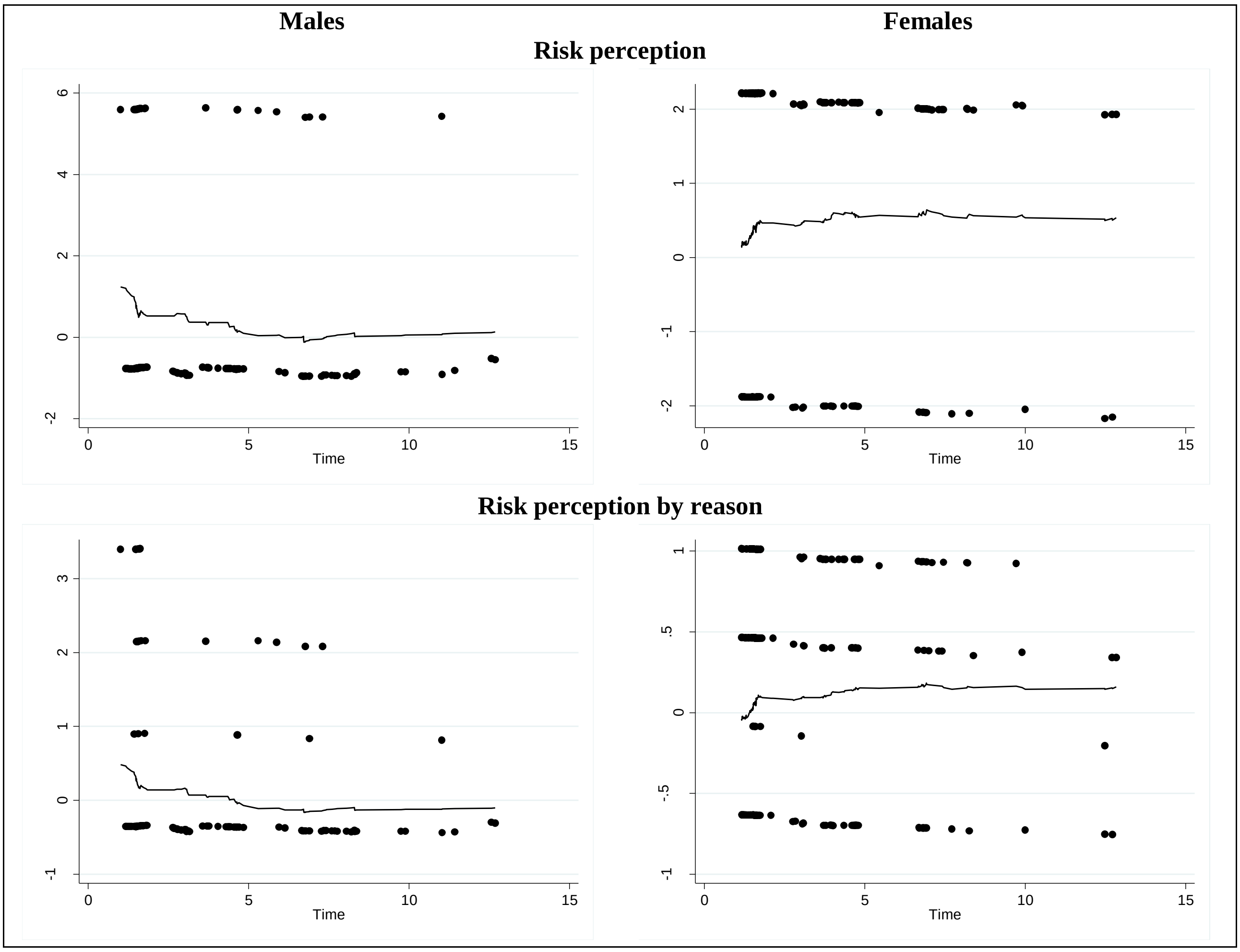

Figure S1: Scaled Schoenfeld residuals over time for risk perception and risk perception by reason, by sex.
